# The pharmacist as a public health resource: Expanding telepharmacy services to address social determinants of health during the COVID-19 pandemic

**DOI:** 10.1016/j.rcsop.2021.100032

**Published:** 2021-06-05

**Authors:** Melanie Livet, Jordana M. Levitt, Alyssa Lee, Jon Easter

**Affiliations:** Center for Medication Optimization (CMO), Division of Practice Advancement and Clinical Education, UNC Eshelman School of Pharmacy, University of North Carolina at Chapel Hill, USA

**Keywords:** Social determinants of health, Telepharmacy, Medication management, COVID, Telehealth

## Abstract

**Background:**

The advent of COVID-19 exacerbated the impact of social determinants of health (SDOH) on patients' ability to manage their health, especially those with chronic conditions. Clinical pharmacists are well positioned to expand the patient care services they already provide to address patients' basic social needs, which may otherwise impede medication access and adherence.

**Objectives:**

The purpose of this exploratory study was to evaluate the feasibility of expanding a comprehensive medication management (CMM) telepharmacy service to include SDOH support. This service was offered as part of four primary care clinics in rural and underserved North Carolina communities. More specifically, the study aimed to describe the expanded service, evaluate stakeholders' experience with the service, and assess short-term impact on patients with diabetes.

**Methods:**

Data collected over the first 4 months of implementation included administrative data used to describe the expanded service; a clinic survey and interviews to assess clinic team members' experience with the service; and patient surveys to evaluate patient satisfaction, as well as impact on SDOH self-efficacy and diabetes quality of life.

**Results:**

Through SDOH screening, the pharmacist identified 26 unresolved COVID-prompted SDOH concerns across 66 patients. These concerns were addressed by the pharmacist through three types of brief interventions, including information provision/education (71%), access to resources (21%), and additional care coordination (7%). Clinic team members perceived the expanded service as highly satisfactory and beneficial*.* Patients also reported high levels of satisfaction and significantly increased their SDOH self-efficacy and diabetes quality of life as a result of the service.

**Conclusion:**

These data provide preliminary insights into the expanded role that pharmacists can play to address current population health gaps that can directly impact patients' engagement with their medication regimen and overall health status.

## 1 Introduction

Effectiveness of medication optimization services are partially dependent on the patient's basic needs being met (e.g., food security, ability to pay for necessities). These social determinants of health (SDOH), defined as “conditions in the places where people live, learn, work, and play that affect a wide range of health and quality-of life-risks and outcomes,”^1^ have been previously found to impact treatment effectiveness and medication adherence.^2^, ^3^, ^4^, ^5^ The advent of COVID-19 only served to exacerbate these challenges, particularly for at-risk patients such as those with chronic disease diagnoses.^6^ Clinical pharmacists are well positioned to expand the patient care services they already provide to address patients' basic social needs, which otherwise may impede medication optimization efforts. Yet, published examples of SDOH being successfully integrated into pharmacy practice are few and far between, with none including effectiveness outcomes thus far.^7^

The purpose of this study was to evaluate the feasibility of expanding a comprehensive medication management (CMM) telepharmacy service to include additional support to address broader patient social concerns brought about by the pandemic. Feasibility was broadly defined as the extent to which a particular service or service component can be *conveniently* and *successfully* carried out in real-world settings.^8^ The CMM telepharmacy service was already being offered by a pharmacist to patients with uncontrolled diabetes in four North Carolina (NC) primary care clinics located in rural and undeserved communities. Of note, the part-time pharmacist was external to the clinics, hired by the project team to deliver CMM as part of a broader 2-year CMM telepharmacy initiative.^9^ For this project, CMM was defined as “a patient-centered approach to optimizing medication use and improving patient health outcomes that is delivered by a clinical pharmacist working in collaboration with the patient and other health care providers.”^10^ More specifically, the pharmacist assesses each medication to determine appropriateness, effectiveness, safety given concurrent therapies, and feasibility to take as intended.

The decision to add the SDOH support component was made in collaboration with the clinics as a result of a 6-month grant awarded by the North Carolina Policy Collaboratory COVID-19 program. The participating clinics were not providing any other formal SDOH screening at the time of this study. More specifically, the service was expanded to include: (1) an SDOH patient assessment to evaluate broader social concerns that might influence their ability to manage their medications; and (2) brief pharmacist-led interventions to address these concerns. The pharmacist used an SDOH screener created by the project team to assess patients' social needs as part of each CMM visit.11., 12., 13. The screener specifically asked about the impact of the pandemic on a number of SDOH (e.g., employment, access to transportation, access to medications), and whether any of the reported SDOH concerns had already been addressed. If the patient noted that the identified concern was still an issue, the pharmacist gathered additional information through discussion with the patient and initiated a brief intervention.

To better understand the potential role that a clinical pharmacist can play to address SDOH, in this case SDOH experienced as a result of the pandemic, this small exploratory study aimed to: (1) describe the expanded support provided by the pharmacist and associated outputs; (2) assess clinic team members' and patients' experience with the service; and (3) generate preliminary effectiveness data by exploring short-term patient reported outcomes.

## 2 Methods

### 2.1 Design and data collection

This exploratory study involved collection of mixed methods data that included administrative indicators, clinic team members and patient surveys, and 30–45 min interviews conducted by the first author with clinic representatives (i.e., the clinic liaisons). “Clinic team members” is defined to include providers and staff (including the clinic liaisons). The clinic liaisons were identified by the clinics, and included a quality assurance (QA) administrator (for three of the participating clinics), and two medical assistants (for one clinic). Table 1 provides an overview of data sources, alignment with the three aims, and data collection details. Selection and groupings of feasibility indicators were guided and adapted from Bowen and colleagues' key areas of focus and potential outcomes for feasibility studies.^14^

**Table 1:** Data overview.

**Aim**	**Data type**	**Indicators**	**Data Source**	**Purpose**	**Collected from**	**Timeline**
Aim 1	Administrative Data	SDOH service implementation progress and outputs (e.g., rates of SDOH interventions)	Spreadsheet	To monitor progress and outputs	Pharmacist	Throughout the implementation period
Aim 2	Surveys	Clinic Satisfaction	Satisfaction Survey (created for study, alpha = 0.96; 3 items; 6-point scale, from “strongly disagree” to “strongly agree”)	To assess satisfaction of the clinic team members with the service	Clinic liaisons, staff and providers (N = 11/16, or response rate = 69%)	After the first 3 months of implementation
		Perceived Benefits	Perceived Benefits Survey^1^ (4 items; 6-point scale, from “strongly disagree” to “strongly agree”)	To assess perceived benefits of the service from the clinics' perspective		
		Service Acceptability	Service Acceptability Measure from IOQ^2^ (7 items; 6-point scale, from “strongly disagree” to “strongly agree”)	To assess acceptability of the service from the clinics' perspective		
		Service Appropriateness	Service Appropriateness Measure from IOQ^2^ (6 items, response range from 1 to 5, from “not at all” to “extremely”)	To assess integration and alignment of the service with the clinic and its practice		
		Service Feasibility	Adapted Service Feasibility Survey from IOQ^2^(7 items; 6-point scale, from “strongly disagree” to “strongly agree”)	To assess practicality of implementing the service		
		Patient Satisfaction/Experience	Adapted Patient Satisfaction/Experience^3^ (Satisfaction: 1 item; 5-point scale, from “very poor” to “excellent”; Experience: 14 items; 5-point scale from "strongly disagree" to "stronly agree")	To assess patient satisfaction and experience with the pharmacist and visits	Patients (N = 10/12 or response rate = 83%)	At end of the implementation period
	Interviews	Clinics' overall experience with the service	Interview transcripts	To assess the clinics' overall experience with the telepharmacy service	Clinic liaisons (N = 3/3 or response rate = 100%)	After the first 3 months of implementation
Aim 3	Surveys	Patient Quality of life	Adapted from Diabetes Quality of Life Survey^4^ (satisfaction with diabetes control: 6 items, 5-point scale, from “very dissatisfied” to “very satisfied”; and adherence to self-care regimen: 3 items, 5-point scale, from “never” to “all the time”)	To assess patients' satisfaction with diabetes control and adherence to self-care regimen	Patients (N = 10/12 or response rate = 83%)	At end of the implementation period
		SDOH Self-Efficacy	SDOH Self-Efficacy survey (created for study, alpha = 0.94) (5 items; 5-point scale, from “strongly disagree” to “strongly agree”)	To assess confidence with SDOH needs being met and ability to problem-solve, access support and resources, and access medications		

1 Venkatesh V, Bala H. Technology Acceptance Model 3 and a Research Agenda on Interventions Subject Areas: Design Characteristics, Interventions. *Decis Sci* [Internet]. 2008;39(2):273–315. doi.org/10.1111/j.1540-5915.2008.00192.x

2 Livet M, Blanchard C, Richard C, et al. Measuring implementation of medication optimization services: Development and validation of an implementation outcomes questionnaire. *Res Soc Adm Pharm.* 2021 (December 2020). doi.org/10.1016/j.sapharm.2021.01.001

3 Shin J, Moczygemba LR, Barner JC, Garza A, Linedecker-Smith S, Srinivasa M. Patient experience with clinical pharmacist services in Travis County Federally Qualified Health Centers. *Pharmacy Practice* 2020 (Apr-Jun);18(2):1751. doi.org/10.18549/PharmPract.2020.2.1751

4 Burroughs J, Mick D. Exploring Antecedents and Consequences of Consumer Creativity in a Problem-Solving Context, Journal of Consumer Research. 2004;31(2):402–411. doi.org/10.1086/422118

### 2.2 Recruitment and implementation

The four primary care clinics participating in this study were initially recruited as part of the broader 2-year CMM telepharmacy initiative^9^ based on a set of pre-selected criteria (e.g., serving rural or underserved communities, reliable internet access). The patients enrolled in the service by the clinics had to meet the following inclusion criteria: HbA1c over 9, at least one additional comorbidity, five or more medications, and at least 18 years of age. The pharmacist connected with the patients in their homes to deliver the scheduled service via video or telephone. Recommended medication changes were communicated to the providers via a pharmacist-initiated electronic health record (EHR) note. Following provider approval, the pharmacist would follow-up with the patient as needed. Pharmacist availability for direct patient care approximated 5 hr/week per clinic. By the end of the grant period, the service had been provided for 4 months, from August to December 2020.

### 2.3 Data analysis

As noted in Table 1, the administrative data were used to identify the number of patients, visits, and social concerns raised by patients, as well as calculate the types of SDOH rates and brief pharmacist-led interventions. A retrospective pre-post survey method was used for all surveys (but the post-only satisfaction and perceived benefits items) to control for response-shift bias.^15^ The surveys completed by the clinic team members were collected 3-month into the implementation phase. Patients completed their surveys at the end of the implementation period reported on in this manuscript (4-month into the implementation phase). Survey data were analyzed using descriptive statistics and *t*-tests^1^ to assess change over time for the relevant indicators (i.e., acceptability, appropriateness, feasibility, diabetes quality of life (QOL), and SDOH self-efficacy). Interview data were analyzed by the first author using content analysis with an a-priori coding structure (i.e., successes, challenges with the service).^16^ Following a first read of the transcripts, subcodes (e.g., successes-benefits for patients) were created and applied during a second read. The analysis was reviewed by the second author, with any disagreements discussed until consensus was reached. IRB approval was obtained prior to data collection. To help offset clinic time and effort, the clinics were provided with a small monetary site incentive as part of the broader CMM telepharmacy initiative. Finally, patients who completed the surveys (i.e., satisfaction, SDOH self-efficacy, and diabetes QOL) received an Amazon gift card.

## 3 Results

### 3.1 Description of the expanded telepharmacy service: SDOH concerns and pharmacist-led brief interventions

Over the 4 months of implementation, the part-time pharmacist conducted 200 telehealth visits with 66 unique patients. One hundred percent of patients (*N* = 66/66) were screened for COVID-prompted concerns. Twenty-seven patients (40.91%) raised 37 concerns or questions related to broader social needs that impacted their ability to manage their diabetes. The types of COVID-prompted SDOH challenges included: employment/income (37.84%, 14/37); health literacy (29.73% (11/37); health behaviors (24.32%, 9/37); transportation (2.70%, 1/37); access to medication (2.70%, 1/37); and insurance status (2.70%, 1/37). Table 2 provides an example of each type of concern. Out of the 37 concerns raised, 11 were reported by the patient as already resolved prior to the screening. The remaining 26 concerns were addressed by the pharmacist through the following types of interventions: educating the patient (71.43%, 20/28); facilitating access to community resources (21.43%, 6/28); and coordinating additional patient care needs with the clinic or community pharmacy (7.14%, 2/28). Table 3 provides examples of specific interventions.

**Table 2:** Types of patient-reported SDOH Challenges.

**Type of SDOH**	**Frequency (%)**	**Example**
Employment/income	14 (37.84)	The patient's hours had been cut due to the pandemic
Health Literacy	11 (29.73)	The patient wanted additional information on COVID
Health behaviors	9 (24.32)	The patient's physical activity was reduced due to gym closures
Transportation	1 (2.70)	The patient did not have transportation to pick up medications (did not want to use public transportation or ride with someone due to COVID)
Access to medication	1 (2.70)	The patient's medication was not available from the manufacturer
Insurance status	1 (2.70)	The patient had lost his health insurance as a result of the pandemic

**Table 3:** Pharmacist-led SDOH Interventions.

**Type of intervention**	**Frequency (%)**	**Example**
Patient education	20 (71.43)	
*Exercising*	2	Shared alternative ways to exercise outside of the gym
*COVID- Testing*	1	Shared expectations for COVID testing
*COVID- Spread/Exposure*	7	Discussed precautions to limit COVID spread in public settings
*COVID- Vaccine*	10	Provided timeline for the patient to receive the vaccine
Community Resources	6 (21.43)	
*Grocery*	1	Introduced the patient to Prime Pantry as a grocery delivery option
*Medication access, affordability*	2	Sent GoodRx coupons to make the patient's medication more affordable
*Occupational*	3	Provided resources to patient to help them find a new job
Care Coordination	2 (7.14)	
*With community pharmacy*	1	Switched patient to a new pharmacy that offers medication delivery
*With primary care clinic*	1	Worked with the clinic to complete patient assistance paperwork to make the patient's medication more affordable

### 3.2 Stakeholders' experience with the expanded service: clinic team members and patients

#### 3.2.1 Clinic team members

Based on survey results, clinic staff and providers across the participating clinics (*N* = 11) expressed high levels of satisfaction (Mean = 5.49(SD = 0.50)) and perceived benefits with the expanded service (Mean = 5.28(SD = 0.54)). In addition, they perceived the service as being successfully implemented based on significant increases in levels of service acceptability (pre-Mean = 4.77(SD = 0.34), post-Mean = 5.39(SD = 0.49), *p* < .01), appropriateness (pre-Mean = 3.79(SD = 0.45), post-Mean = 4.38(SD = 0.55), p < .01), and feasibility (pre-Mean = 4.48(SD = 0.48), post-Mean = 5.18(SD = 0.60), p < .01) from baseline to 3 months post-implementation. Demographics for clinic team members are presented in Table 4.

**Table 4:** Clinic and patient demographics.

**Clinic** team members **characteristics (N = 11)**	**Frequency (%)**
Role in the project	
Staff (who were not Clinic Liaisons)	3 (27.27)
Provider	5 (45.45)
Clinic Liaison	3 (27.27)
Gender	
Male	4 (36.36)
Female	7 (63.64)
Race	
Black or African American	1 (9.09)
White	8 (72.73)
Multi-Racial	2 (18.18)
Other	
Highest Level of Education	
Bachelor's	3 (27.27)
Associates	1 (9.09)
MD	4 (36.36)
MS/MA	3 (27.27)
Years of Experience at the Clinic	
0–4 years	6 (54.55)
5–9 years	1 (9.09)
10–14 years	0 (0.00)
15 to 19 years	1 (9.09)
20+ years	3 (27.27)
Role in the project	
Staff (who were not Clinic Liaisons)	4 (36.36)
Provider	5 (45.45)
Clinic Liaison	2 (18.18)


**Patient Characteristics (N=10)**	**Frequency (%)**

Age	
25–34	2 (20.00)
35–44	1 (10.00)
45–54	6 (60.00)
75 or older	1 (10.00)
Gender	
Male	5 (50.00)
Female	5 (50.00)
Race	
Black or African American	5 (50.00)
White	3 (30.00)
Multi-Racial	1 (10.00)
Other	1 (10.00)
Ethnicity	
Hispanic or Latino(a)	2 (20.00)
Not Hispanic or Latino(a)	8 (80.00)

The qualitative information obtained through the interviews with the clinic liaisons (*N* = 3) further validated survey results. The pharmacist was praised for her ability to interact positively with the providers (“all spoke very positively in the fact that the communication is good”); the service scope was deemed highly appropriate (“[clinic] likes the fact that it was beyond just the medications [to include health coaching and SDOH screening]”); and the service was perceived as highly beneficial to both patients and providers. Identified benefits to patients included extra attention from a healthcare professional, dedicated time to optimize medications, and consideration of the SDOH that might impact their health (“that's great that it [the SDOH component] was added on because it builds that relationship and their [patients'] sense of confidence, which ultimately will affect how they are doing on their medication regimen”). Identified benefits for the providers included more efficient provider visits, and extra support (“the fact that we can provide an additional support layer where they [physicians] don't have to have all of the answers and they've got that sense of team and help”). The only challenge identified with the expanded service was the lack of reimbursement mechanism for the SDOH component of the service.

#### 3.2.2 Patients

Based on the patient satisfaction survey, patients (*N* = 10) reported the highest level of satisfaction possible with their pharmacist (Mean = 5.0 (SD = 0.00)). They also reported a positive experience with the telepharmacy visits, including their interaction with the pharmacist, the quality of information they were provided, the level of support for self-care, and their level of involvement with decisions made about their medications (Mean = 4.91(SD = 0.14)). Patients described the pharmacist as knowledgeable, informative, friendly, and caring. Demographics for participating patients are presented in Table 4.

### 3.3 Preliminary effectiveness: short-term patient-reported outcomes

On the SDOH self-efficacy patient survey, patients reported increased confidence with having their social needs met, being able to access needed resources and support, and being able to access their medications after meeting with their pharmacist (pre-Mean = 3.42(SD = 0.90), post-Mean = 4.40(SD = 0.78), *p* < .01). Similarly, on the Diabetes QOL patient survey, patients reported moderate satisfaction with diabetes control prior to meeting with the pharmacist (Mean = 3.57(SD = 0.67)). After at least two telepharmacy visits, patients' satisfaction with diabetes control significantly increased (Mean = 4.52(SD = 0.57), *p* < .01). Similarly, scores on the adherence to self-care regimen subscale significantly increased after two visits with the pharmacist (pre-Mean = 3.47(SD = 0.59), post-Mean = 4.07(SD = 0.60), p < .01).

## 4 Discussion

Gaining insights into the role that pharmacists can play to address SDOH as part of direct patient care is critical to understanding their value as a public health resource. Ultimately, the ability to address these factors has significant implications for ensuring medication access, assisting patients with medication non-adherence, and improving health outcomes.^2^, ^3^, ^4^, ^5^ With physicians already overburdened, pharmacists shouldering this additional responsibility presents an opportunity to advance pharmacy practice and contribute to population health.

Results from this study contributes to the emerging literature supporting pharmacists as effective healthcare extenders working outside of the traditional boundaries. More specifically, it provides insights into the feasibility of integrating SDOH screening and brief interventions into a CMM telepharmacy service that was already offered to patients with diabetes as part of four primary care clinics.

Worth highlighting are three key findings and their implications. First, beyond initial screening, the types of interventions delivered by the pharmacist to assist patients with SDOH concerns tended to fall into three main categories: information provision/education, access to resources, and additional care coordination. These findings are consistent with previous literature on SDOH emphasizing the need to present the patient with relevant information, understand the patient's community and connect them to existing resources, and address the SDOH from a team-based approach.^17^^,^^18^ Considering inclusion of these types of brief interventions might be useful when designing formal SDOH programs that can easily be integrated into an existing pharmacist-led service.

Second, both clinic team members and patients perceived the expanded service as highly satisfactory and beneficial. Clinic providers and staff endorsed the service, reported it to align well with their practices and patient care approach, and deemed it feasible to carry out. The pharmacist was perceived to be seamlessly integrated as part of the clinic teams and able to build a relationship of trust with the patients. Positive service perceptions and a highly relatable healthcare professional (i.e., pharmacist) have been previously found to be essential conditions for successful integration of SDOH into clinical services.^18^, ^19^, ^20^ These implementation facilitators should be considered prior to service delivery and included as part of any implementation roadmap. Selection of a pharmacist with strong interpersonal skills in addition to clinical expertise seems essential for what could be particularly sensitive patient interactions.^21^^,^^22^ Likewise, use of implementation strategies to ensure stakeholder buy-in and influence perceptions of the SDOH service (e.g., ensure alignment of the service with their needs, use stakeholder feedback to inform decision-making) needs to be part of the planning and implementation process.^23^, ^24^, ^25^, ^26^

Third, although preliminary, there was evidence that the expanded service had a positive impact on patients' SDOH self-efficacy and self-reported diabetes QOL (including diabetes control and adherence to self-care regimen). In other words, by combining a CMM service with simple yet practical interventions designed to assist the patients with immediate COVID-prompted SDOH concerns that may interfere with their medication therapy, the pharmacist was able to improve patient outcomes. Although it is premature to conclude on the utility of integrating SDOH support with a medication management intervention, these results are promising, especially in light of the sparse literature in this area. As SDOH become an area of focus in healthcare, identifying ways to assist patients with broader social needs as part of routine care is critical. Results from this study provide evidence that pharmacists can effectively help address SDOH with minimal disruptions to their patient care workflows.

Given its exploratory nature, this study is not without its limitations. First, the small sample size limits generalizability of findings, beyond the context of this project. Additional research is needed to provide further evidence in support of the study results. Second, the implementation period was relatively short. Although these data are encouraging, additional findings may have emerged had the service been implemented for a longer period of time. Third, although promising, evidence of the intervention effectiveness needs to be further validated using more stringent research designs. Finally, the SDOH service was combined specifically with CMM and delivered by one clinical pharmacist as part of a telepharmacy pilot for patients with uncontrolled diabetes. Insights from this study need to be further validated using diverse combination of services, delivery modalities (e.g., in-person), and pharmacists within other healthcare contexts.

## 5 Conclusion

In summary, these results provide preliminary insights into the expanded role that pharmacists can play to address current population health gaps that can directly impact patients' engagement with their medication regimen and overall health status.

## Funding

Funding for this project was awarded by the North Carolina Policy Collaboratory. The broader CMM telepharmacy initiative is funded by the Eshelman Institute for Innovation at the UNC Eshelman School of Pharmacy.

## Declaration of Competing Interest

The authors declare that they have no known competing financial interests or personal relationships that could have appeared to influence the work reported in this paper. Funding sources are reported above.

## Footnotes

1 Because of the small sample size, the Wilcoxon rank test (non-parametric) was also conducted determine whether the difference between paired observations was significant from baseline to post-intervention for the relevant surveys. Because results were similar regardless of test (Wilcoxon or *t*-tests), only *t*-tests are reported.
